# Supplementary material for: PTPN2 copper-sensing relays copper level fluctuations into EGFR/CREB activation and associated CTR1 transcriptional repression
Source: Nat Commun. 2024 Aug 13;15:6947. doi: 10.1038/s41467-024-50524-5 (PMC11322707; doi:10.1038/s41467-024-50524-5)
Supplement: Supplementary file 1 — Supplementary Information [file 41467_2024_50524_MOESM1_ESM.pdf]

PTPN2 copper-sensing relays copper level fluctuations into EGFR/CREB activation and associated *CTR1* transcriptional repression

M. O. Ross, et al.

Supplementary Information

Example qPCR Data Analysis Code

```
# Load packages
library(dplyr)
library(tidyverse)
library(readxl)
library(ggprism)

# Generate a box plot with datapoints and mean defined by “x”
print(ggplot(subset.df, aes(fill = Incubation_time, y = Fold_Change, x = Incubation_time)) +
        geom_dotplot(aes(as.factor(Incubation_time)),
                     binaxis = "y",
                     stackdir = "center",
                     position = position_dodge(width = 0.85),
                     binwidth = 0.015,
                     alpha = 1) +
        geom_boxplot(aes(factor(Incubation_time)),
                     position = position_dodge(width = 0.85),
                     outlier.colour = NULL,
                     alpha = 0.8,
                     lwd = 0.7) +
        stat_summary(
          aes(factor(Incubation_time)),
          position = position_dodge(width = 0.85),
          fun="mean",
          color="black",
          shape=4) +
        theme_classic() +
        theme(text = element_text(size=27),
              plot.title = element_text(hjust = 0.1, vjust = -2, color = "black", size = 27, face = "bold"),
              axis.ticks.length  = unit(0.5, "cm"),
              axis.ticks = element_line(colour = "black", size = 2),
              axis.line = element_line(colour = 'black', size = 2),
              axis.text.x = element_text(colour = "black", size = 27),
              axis.text.y = element_text(colour = "black", size = 27),
              legend.position = "none") +
        scale_fill_manual(values = c("0" = "#C6E1ED",
                                     "10" = "#3795BE")))

## Calculating significance values for all of our different conditions and turning them into a dataframe
# Extract p-value for a given transcript, concentration of copper, and timepoint, and place into dataset
calc_p <- function(DATASET) {
  transcripts <- levels(DATASET$Transcript) # making a vector of all of our transcripts
  concs <- levels(DATASET$Conc) # making a vector of all of the copper concentrations
  mins <- levels(DATASET$Time) # making a vector of all of the time points
  p_list <- c() # making an empty list where p-values will be stored
  t_list <- c() # making an empty list where transcript will be stored
  c_list <- c() # making an empty list where concentrations will be stored
  m_list <- c() # making an empty list where time point will be stored
```

```
# looping for each transcript
  for (i in 1:length(transcripts)) {

# looping for each time point
    for (y in 1:length(mins)) {
    DF <- DATASET %>% # filtering our dataset for transcript and time of interest
      filter(Transcript == transcripts[i] & # so we only calculate p-values within that condition
             Time == mins[y])

# iterating over each Cu conc to generate a diff expression p-value in comparison to 0µM
      for (x in 2:length(concs)) {
        if (count(filter(DF, Conc == concs[x])) >= 2) {
        p <- t.test(DF$Fold_Change[DF$Conc == "0"],
                    DF$Fold_Change[DF$Conc == concs[x]])$p.value
        p_list <- c(p_list, p)
        c_list <- c(c_list, concs[x])
        t_list <- c(t_list, transcripts[i])
        m_list <- c(m_list, mins[y])
     }
    }
   }
  }
  final_dataframe <- data.frame(t_list, c_list, m_list, p_list)
  return(final_dataframe)
}

# Make dataframe more legible MORE LEGIBLE
p_value_df <- function(DATASET) {
  df <- calc_p(DATASET)
  df <- df %>%
    rename("Transcript" = t_list,
          "Concentration of Copper" = c_list,
          "Time" = m_list,
          "t-test p-value" = p_list)
  return(df)
}
A549_p_values <-  write.csv(p_value_df(A549), "A549 p-values.csv", quote=FALSE)


## Exporting the avg and std dev data
A549_group <- A549 %>% group_by(Conc, Time, Transcript) %>%
mutate(AVG = mean(Fold_Change, na.rm = TRUE),
         STD = sd(Fold_Change, na.rm = TRUE)) %>%
  filter(Conc != 0) %>%
  ungroup()
```

<u>Example GTEx Data Processing (Psuedo)Code</u>

```
#packages
library(ggpubr)
library(DESeq2)

#read in Annotations
sample.df <- read.delim("GTEx_Analysis_v8_Annotations_SampleAttributesDS.txt", as.is=TRUE,
header=TRUE, row.names=1)

#Call RNA-seq samples that pass analysis freeze
rnaseq.sample.df <- sample.df[sample.df['SMAFRZE']=='RNASEQ', ]

#Summary of Tissues
as.matrix(sort(table(rnaseq.sample.df['SMTSD']), decreasing=TRUE))

#Call a specific tissue
Tissue.sample.df = rnaseq.sample.df[rnaseq.sample.df['SMTSD']=='Tissue - Identifier', ]
head(Tissue.sample.df)

#Load expression data
Counts.df <- read.delim("GTEx_Analysis_2017-06-05_v8_RNASeQCv1.1.9_gene_reads.gct.gz",
                as.is=T, row.names=1, check.names=FALSE, skip=2)
gene.names.df <- Counts.df[, 'Description', drop=FALSE]
Counts.df <- Counts.df[, !(names(Counts.df) %in% c('Description'))]

cat(paste("Number of genes in table: ", dim(Counts.df)[1]))

#Call donor IDs from tissue and call tissue specific expression counts
transpose.Counts.df <- t(Counts.df)
donor.ids <- rownames(Tissue.sample.df)
Tissue.Counts.df <- transpose.Counts.df[rownames(transpose.Counts.df) %in% donor.ids, ]

#Export unnormalized counts
write.csv(Tissue.Counts.df, "unnormalized.Tissue.counts.csv")

#load count matrix and info table containing samples split into 2 arbitrary conditions
dat <- t(read.csv("unnormalized.Tissue.counts.csv", header = T, row.names = 1))
info <- read.table("Tissue_colData.txt", header = T, sep = '\t')
dds <- DESeqDataSetFromMatrix(dat, info, ~Condition)

#remove lowly expressed genes
keep <- rowSums(counts(dds)) > “Total number of samples”*2
dds <- dds[keep,]

#main DESeq
ddsDE <- DESeq(dds)

#export normalized read counts
normCounts <- counts(ddsDE, normalized = T)
write.csv(normCounts, "normal.Tissue.csv")

#transpose matrix & convert matrix to dataframe
transpose_Tissue <- t(Tissue)
```

Tissue.df <- as.data.frame(transpose_Tissue)

#correlate Transcript_1 vs. Transcript_2

```
pdf(file = "Tissue_Transcript_1_vs_Transcript_2.pdf", width = 10, height = 10)
ggscatter(Tissue.df, x = "ENSG_ID_Transcript_2", y = " ENSG_ID_Transcript_1", alpha = 0.5, col =
"blue3", size = 4,
          xlab = "TRANSCRIPT_2", ylab = "Transcript_1", add = "reg.line", add.params = list(size = 6),
conf.int = TRUE,
          cor.coef = TRUE, cor.method = "pearson", pch = 20) +
  theme(axis.ticks = element_line(size = 2.5), axis.line = element_line(size = 3.0),
        axis.ticks.length = unit(0.6, "cm"))
dev.off()
```

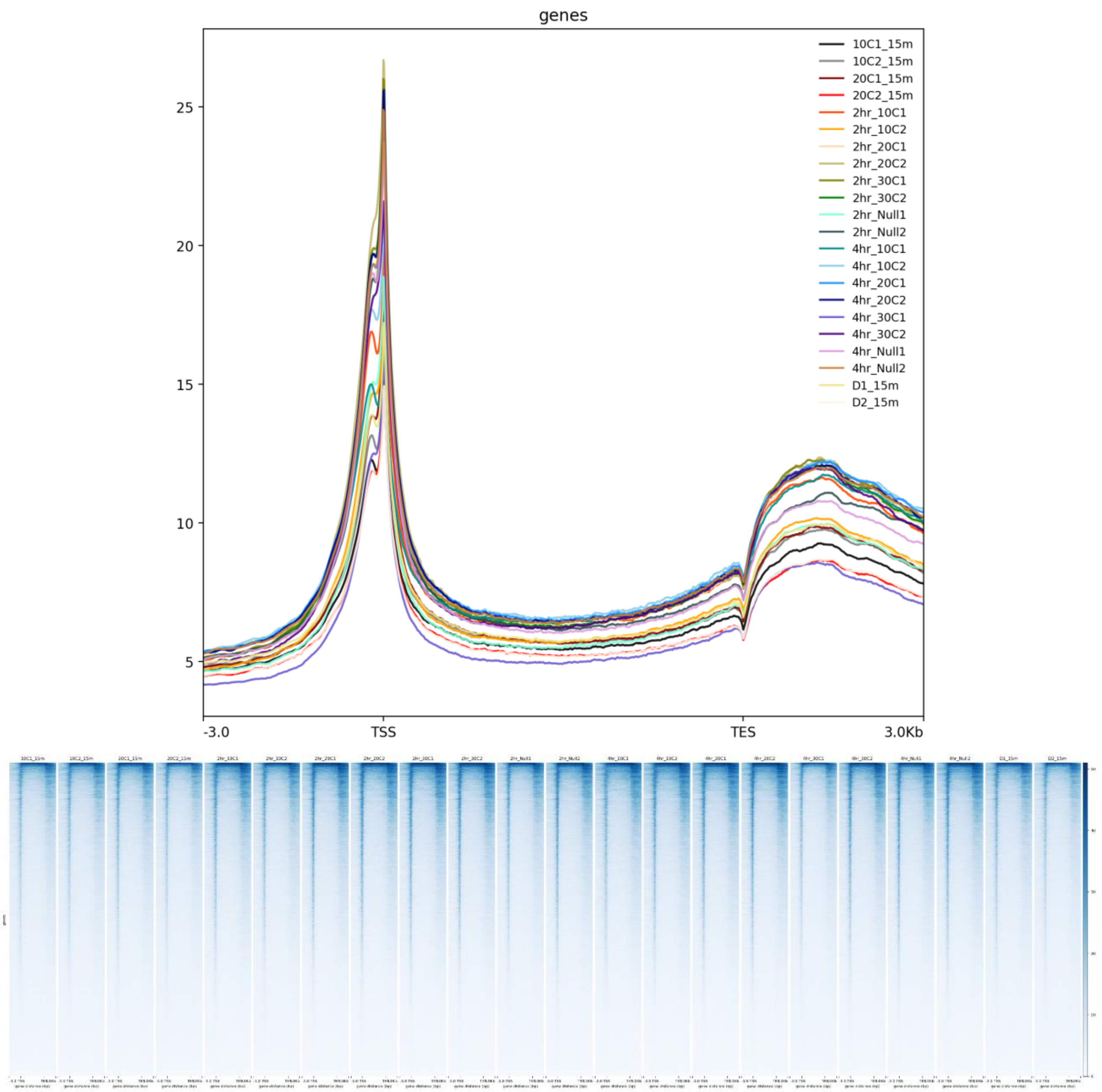

Supplementary Figure 1. **A549 KAS-seq heatmap and profiles.** (*top*) Heatmap and (*bottom*) profiles of A549 KAS-seq data, with individual replicates shown separately. The KAS signal shows clear enrichment near the transcription start site (TSS), and to a lesser extent, near the transcription end site (TES). Labels denote the conditions probed for each individual replicate (for example, 10C1_15m was replicate 1 of A549 cells supplemented with 10 μM $CuCl_2$ for 15 minutes, 10C2_15m was replicate 2 of the same condition, etc.).

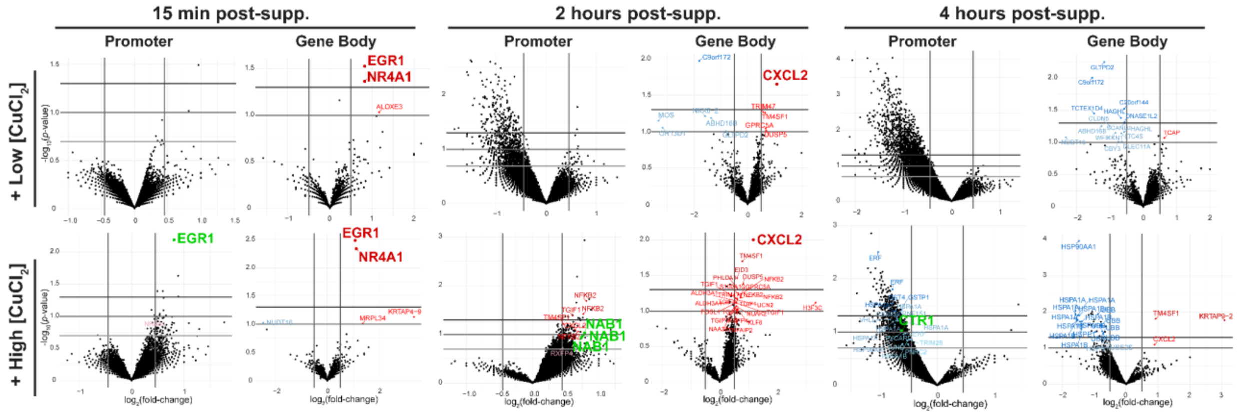

Supplementary Figure 2. **Copper-supplemented A549 KAS-seq volcano plots.** Volcano plots compared copper supplemented to unsupplemented cells. Significantly upregulated gene bodies/promoters post-copper supplementation in red, downregulated in blue, and select promoters (*EGR1*, *NAB1* and *CTR1*) in green. *EGR1*, *NR4A1*, *CXCL2, NAB1,* and *CTR1* shown in bold. Labeled promoter data points correspond to up-/down-regulated gene bodies at that condition and timepoint. Heavy horizontal lines denote p-value = 0.2 (promoter only), 0.1, and 0.05, heavy vertical lines denote $|\log_2$fold-change$|$ = 0.45 (promoter) or 0.5 (gene body). The multiple *NAB1* promoter datapoints correspond to different promoter region boundaries. For clarity, ubiquitin and heat shock protein differential ssDNA levels omitted from 4-hour high Cu gene body responses (which comprise the unlabeled, significantly repressed data points). In the high Cu datasets, there is excellent agreement for activated/repressed gene bodies with corresponding promoter responses, but not vice versa; promoter KAS-seq measurements are far more sensitive (Supplementary Fig. 1). n = 2 biological replicates for each condition.

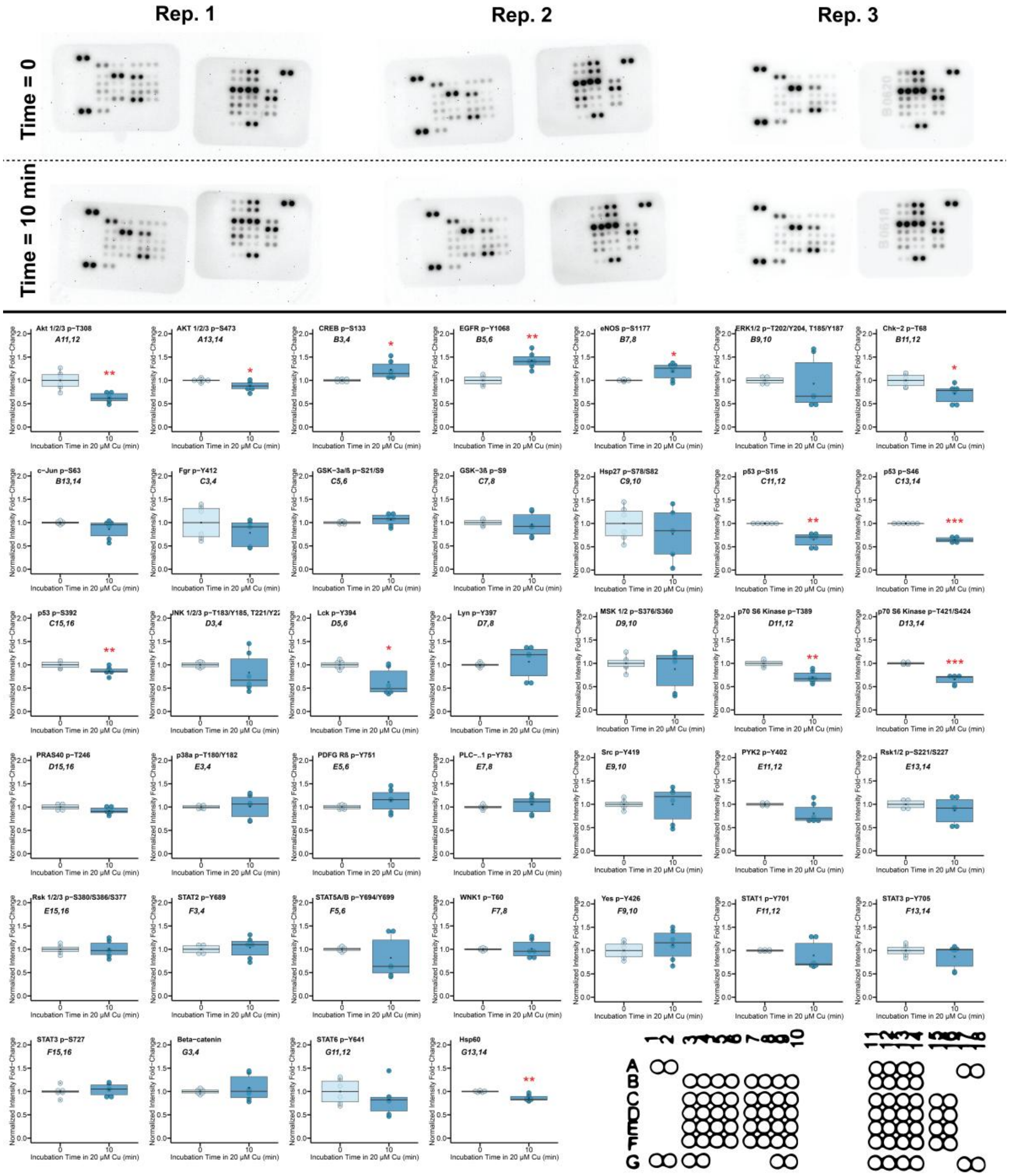

Supplementary Figure 3. **Copper-stimulated phosphorylation patterns.** (*top*) HEK 293T proteome profiler phospho-kinase antibody arrays, (*bottom*) integrated pixel densities reporting chemiluminescence intensity for treatments with or without supplementation of 20 μM $CuCl_2$ for 10 minutes (in cell culture incubator: 37°C, 5% CO_2, 90% humidity). Graph titles indicate the protein name and phosphorylation site (residue) reported. Italicized letters below name of phosphorylation site in graphs corresponds to the location of the antibodies on the membrane array, as indicated by the key in the bottom right. Antibody locations A1,2; A17,18, and G1,2 are reference (control) spots, while G9,10 and G17,18 are negative controls (PBS). n = 3 biological replicates for each condition, where each biological replicate contained antibodies spotted in duplicate. Same box plot definitions as in Figure 1.

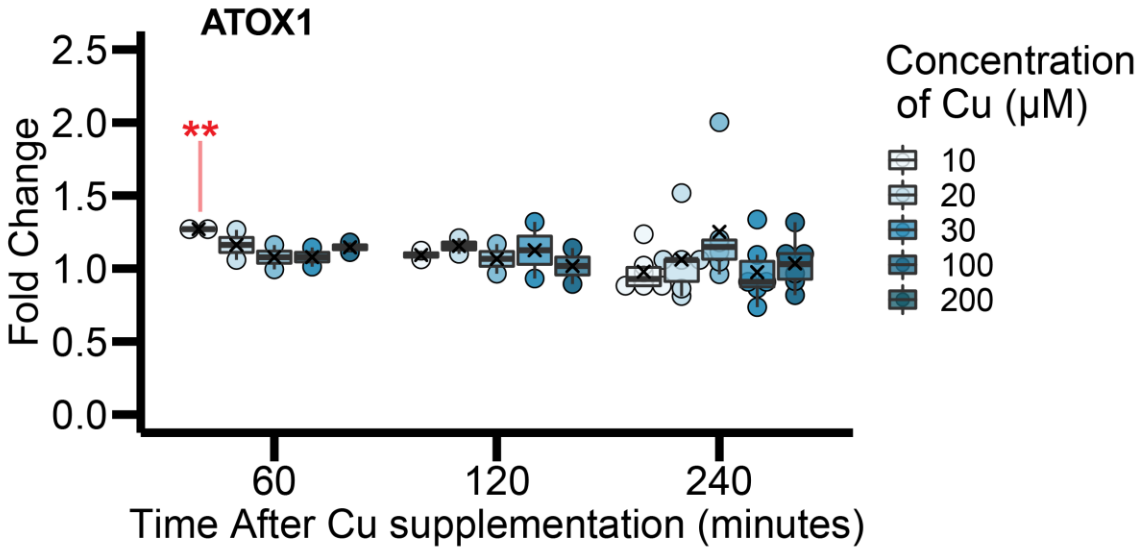

Supplementary Figure 4. **Copper-stimulated changes in A549 *ATOX1* expression levels.** RNA-qPCR results of RNA extracted from A549 cells following supplementation with a screen of $CuCl_2$ concentrations for the times indicated on the X-axis. As expected, there were no significant changes following $CuCl_2$-supplementation, except for the one anomalous condition after 60 minutes at the lowest $CuCl_2$ supplementation level. Individual datapoints correspond to biological replicates. Same box plot definitions as in Figure 1. *n* = 2, 2, 6, for 60 min, 120 min, and 240 min post-Cu, respectively. Source data are provided as a Source Data file, as are exact *p*-values.

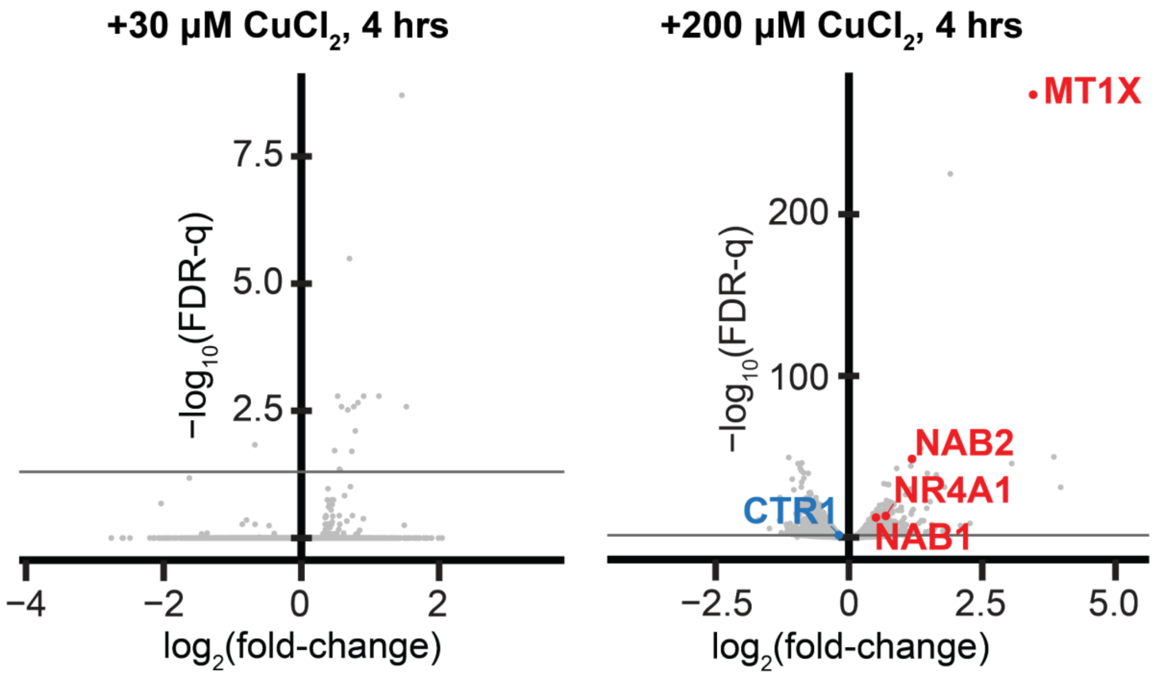

Supplementary Figure 5. **Copper-supplemented A549 RNA-seq volcano plots.** Plots were generated from cells supplemented with $CuCl_2$ for 4 hours relative to unsupplemented cells. Select transcripts with significantly altered expression patterns post-Cu treatment are labeled with standard nomenclature (blue for downregulated, red for increased expression). No transcripts of interest were significantly altered by 30 μM $CuCl_2$ treatment for 4 hours. Gray horizontal line denotes FDR-q = 0.05. FDR-q is false discovery rate corrected p-value. The decrease in *CTR1* expression levels 4 hours post-supplementation with 200 μM $CuCl_2$ is statistically significant (FDR-q < 0.05). $n = 3$ biological replicates for each condition.

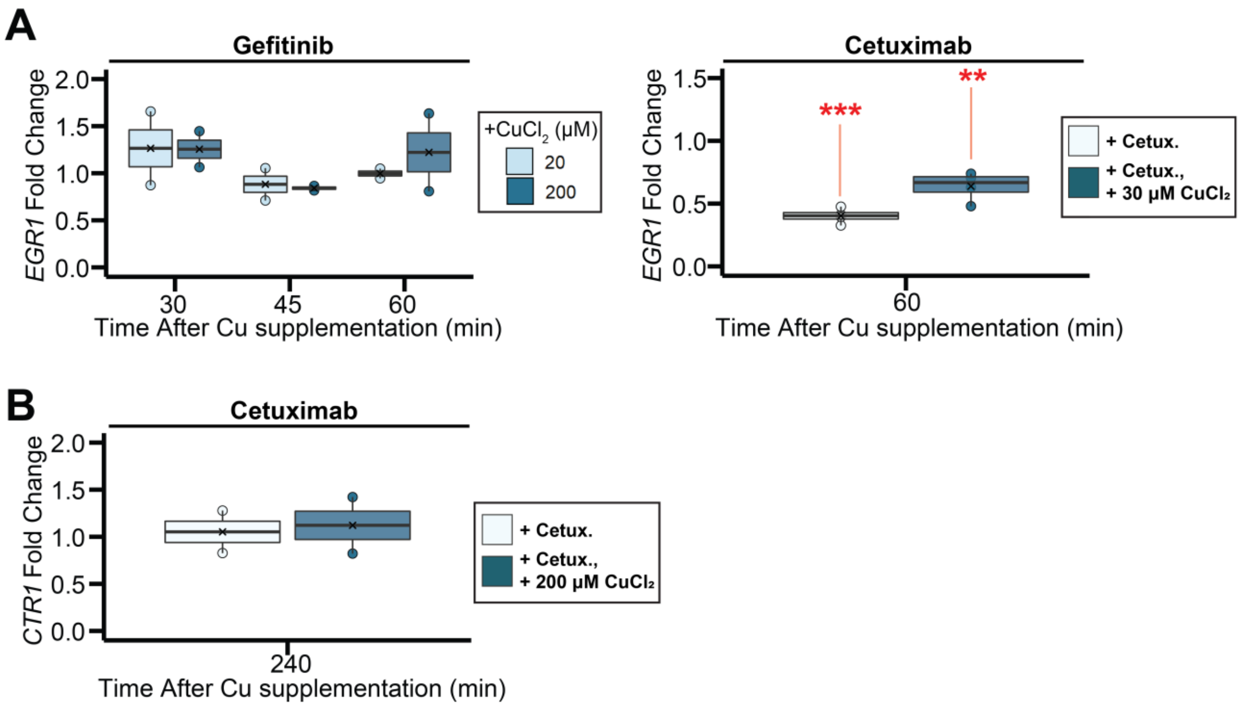

Supplementary Figure 6. **Effects of EGFR inhibitors on copper-stimulated changes in *EGR1* and *CTR1* expression levels.** RNA-qPCR of **A** *EGR1* and **B** *CTR1* transcript levels following EGFR inhibitor pre-treatment, and subsequent $CuCl_2$ or no addition (control). Data points correspond to biological replicates. *EGR1* levels were unaltered following copper-treatment of gefitinib (an EGFR inhibitor compound)-pretreated cells and were lower in copper-treated, cetuximab (an EGFR inhibitor monoclonal antibody)-pretreated cells relative to untreated cells. *CTR1* levels, conversely, were unchanged in cetuximab pre-treated cells supplemented with $CuCl_2$. Collectively, these results validate copper-driven activation of MAPK/ERK signaling and associated transcriptional activities, as well as copper-stimulated *CTR1* transcriptional repression. Same box plot definitions as in Figure 1. *n* = 2, 4, and 2 biological replicates for gefitinib pretreated *EGR1* fold-change, Cetuximab pre-treated *EGR1* fold-change, and Cetuximab pre-treated *CTR1* fold-change. Source data are provided as a Source Data file, as are exact *p*-values.

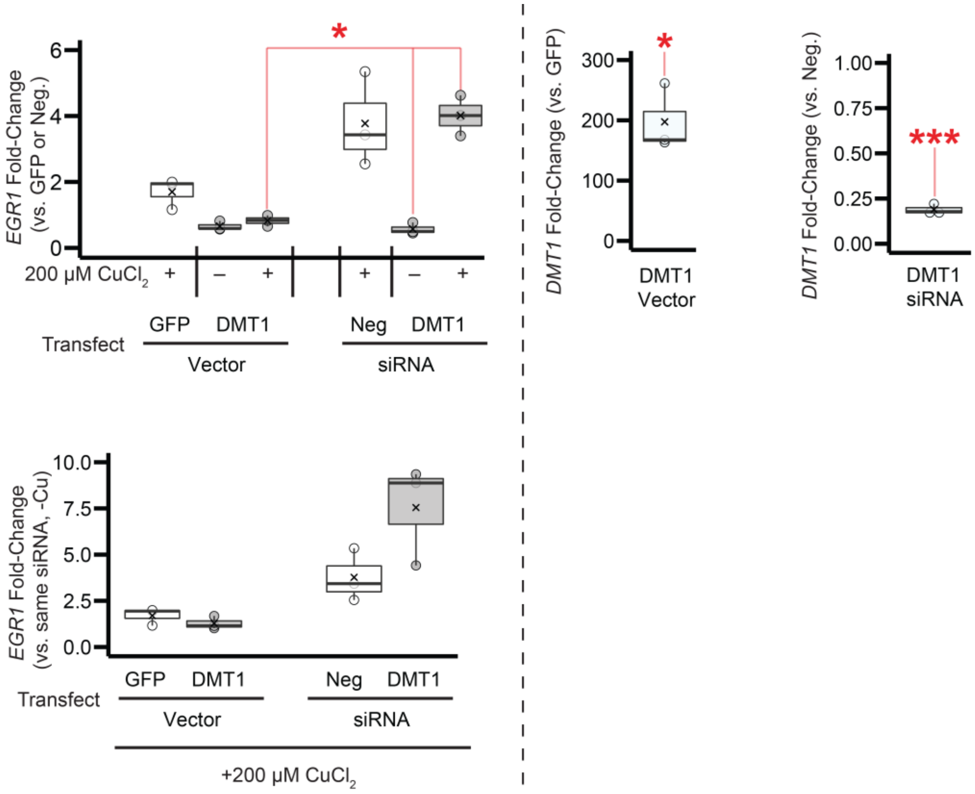

Supplementary Figure 7. **Effects of modulating DMT1 expression via siRNA knockdown or expression vector transfection, with alternative interpretations.** (left, top) Fold-changes are reported relative to the dsRed-GFP vector transfected (vector samples) or negative control siRNA transfected (siRNA samples) without $CuCl_2$ supplementation. (left, bottom) Each result is reported as the fold-change between the denoted siRNA knockdown after 60 min 200 μM $CuCl_2$ supplementation and the siRNA knockdown by itself. (n = 3 replicates per condition). (right) DMT1 expression fold-changes reported for the same samples as on the left side. Same box plot definitions as in Figure 1. Source data are provided as a Source Data file, as are exact p-values.

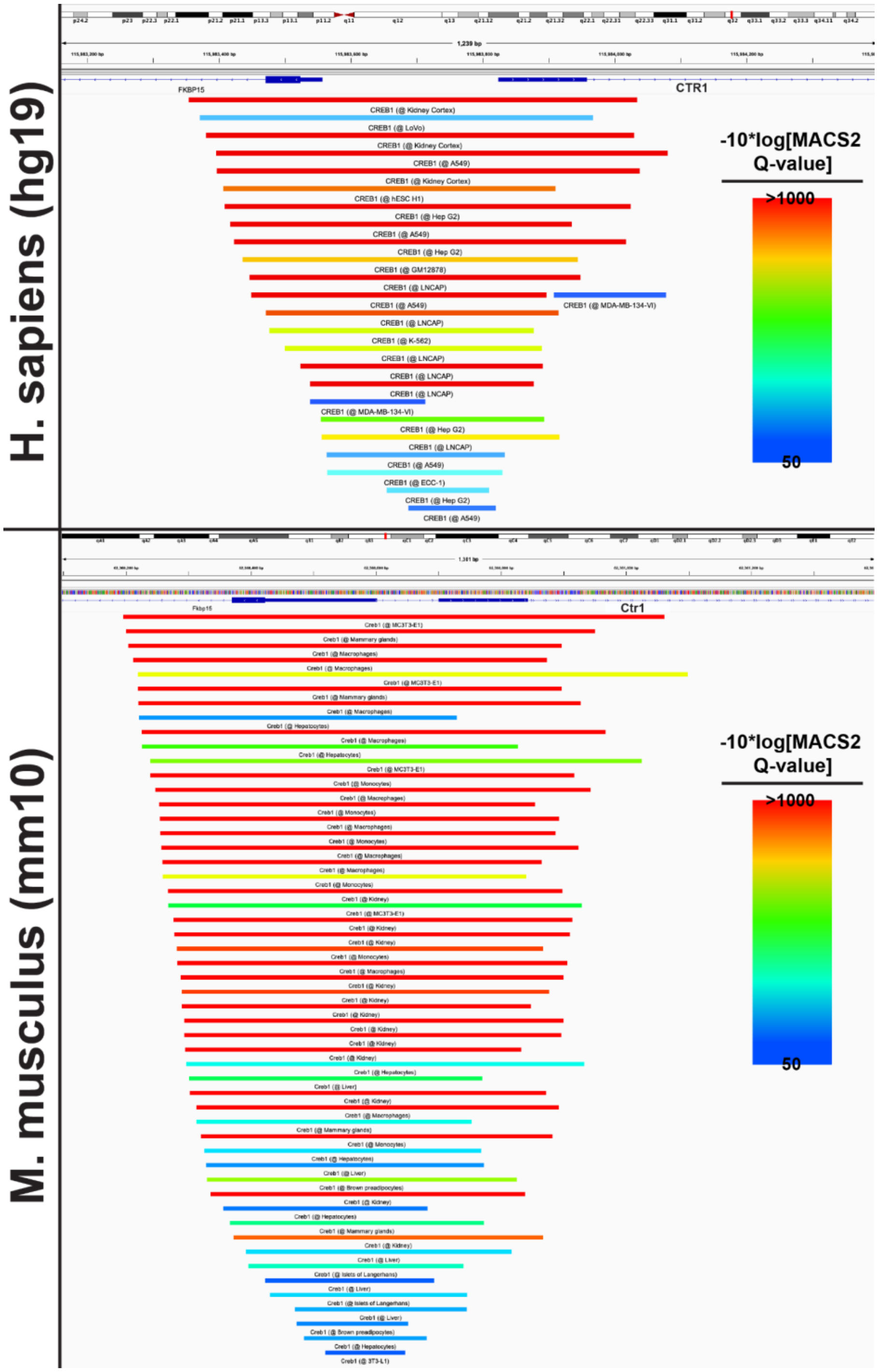

Supplementary Figure 8. **All CREB1 ChIP-Atlas results.** Bed file summary of all *Homo sapiens* (genome build hg19) and *Mus musculus* (genome build mm10) CREB1/Creb1 ChIP-seq experiments in the ChIP-Atlas, corresponding to CREB1 binding in the *CTR1*/*Ctr1* promoter. 22 of 31 total human and 52 of 81 total mouse ChIP-seq experiments identified significant binding in the *CTR1*/*Ctr1* promoter, as shown above. MACS2 Q-value denotes the FDR-adjusted *p*-value.

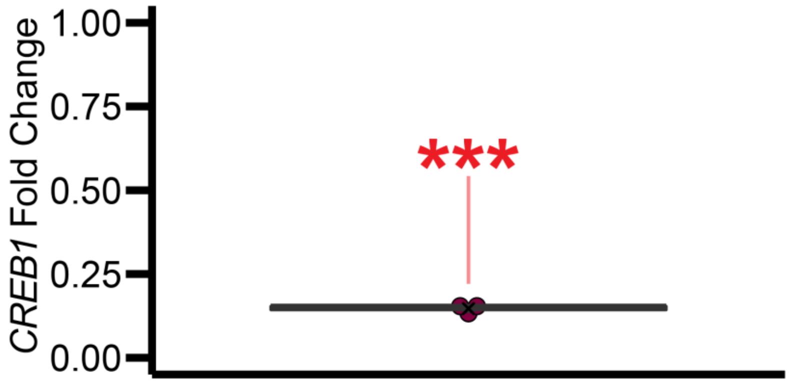

Supplementary Figure 9. **Validation of CREB1 knockdown following *CREB1* siRNA transfection.** *CREB1* transcript expression fold-change following CREB1 siRNA knockdown in A549 cells (relative to negative control siRNA transfection) for $n = 3$ biological replicates. Same box plot definitions as in Figure 1. Source data are provided as a Source Data file, as are exact p-values.

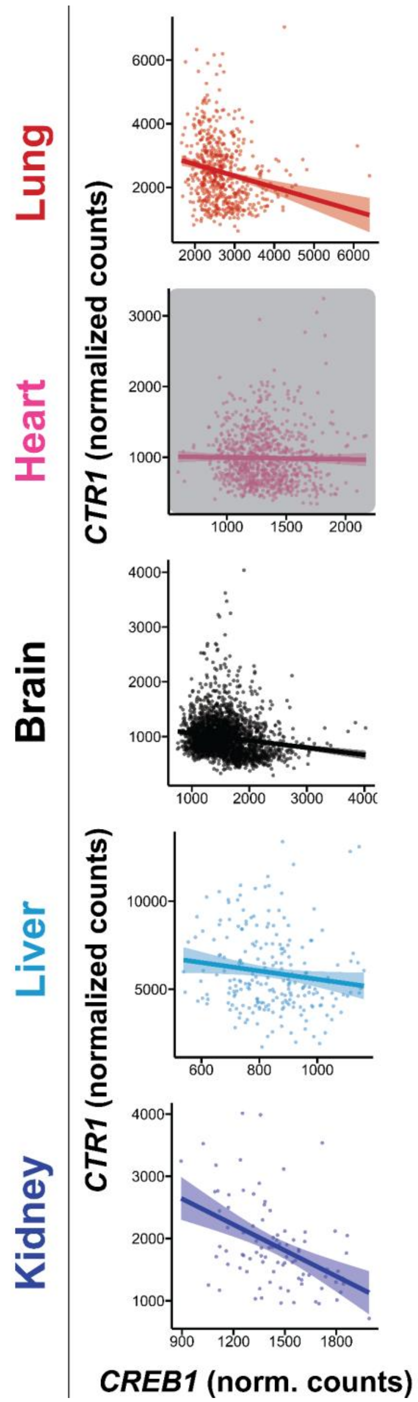

Supplementary Figure 10. ***CTR1/CREB1* RNA-seq expression value correlations.** Correlation of *CTR1* transcript expression levels with those of *CREB1* from GTEx RNA-seq datasets across the five vital organs. The statistically insignificant correlation in the heart tissue is covered by a gray box. Sample sizes are the same is in Fig. 3. norm. counts is normalized counts.

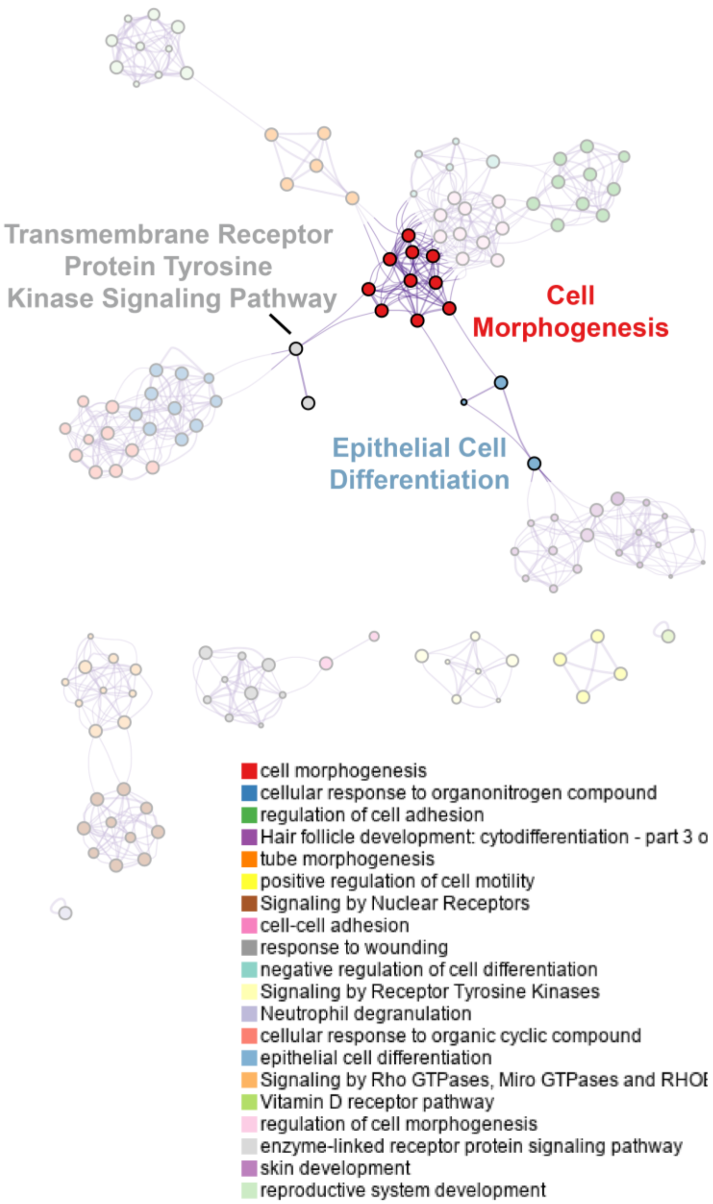

Supplementary Figure 11. **Enriched pathways/process network generated from transcripts significantly downregulated by TM treatment of MDA-MB-468 cells.**

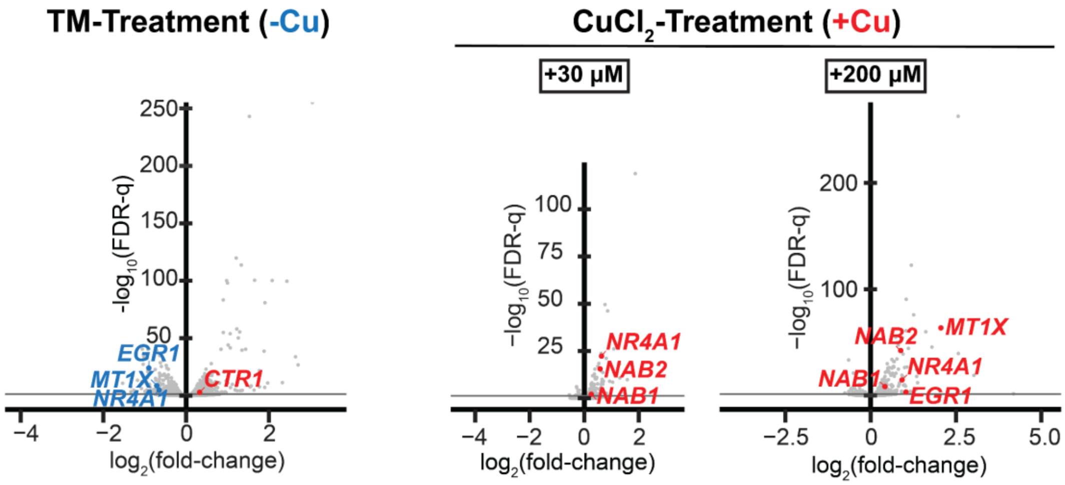

Supplementary Figure 12. **Cu- or TM-supplemented MDA-MB-468 RNA-seq volcano plots.** Full volcano plots depicting differential expression results from TM-treated MDA-MB-468 RNA-seq, as well as $CuCl_2$-treated A549 cells for 2 hours, as featured in Fig. 2. Gray horizontal line denotes FDR-q =0.05. *n* = 2 for TM, 3 for Cu: biological replicates for each condition. FDR-q is false discovery rate corrected p-value.

A

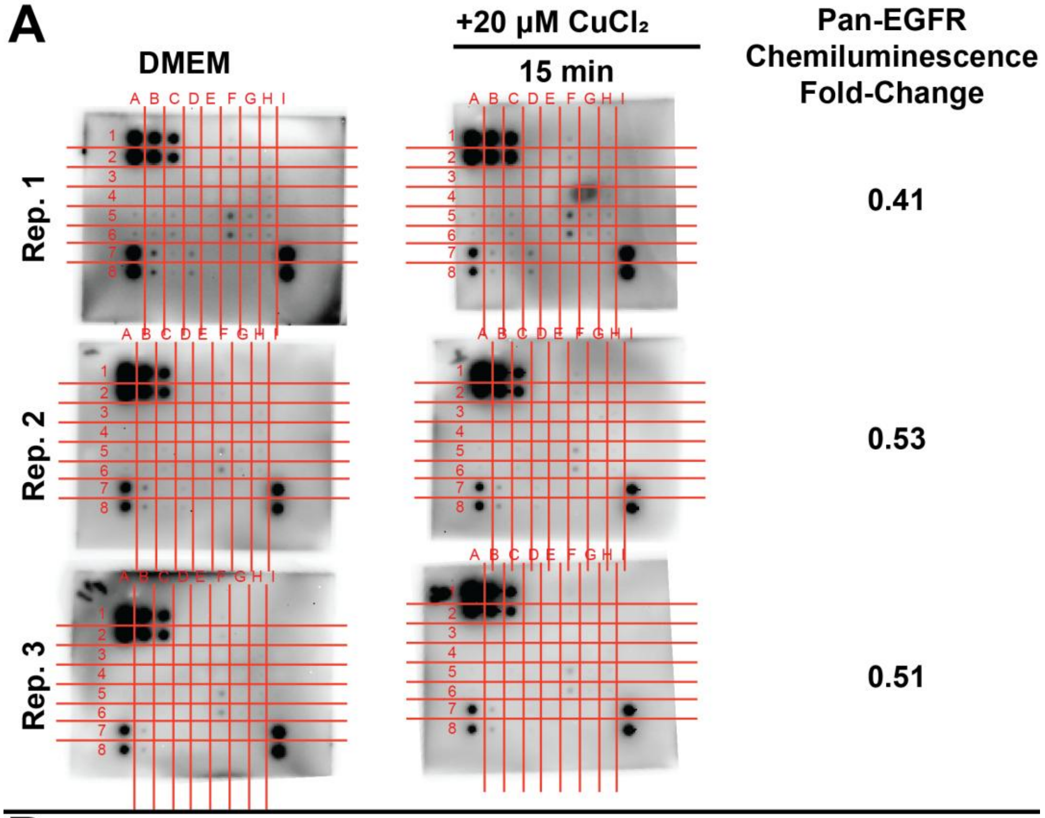

B

	A	B	C	D	E	F	G	H	I
1	P1<br>A1	P2<br>B1	P3<br>C1	Blank<br>D1	Neg<br>E1	EGFR (Tyr845)<br>F1	EGFR (Tyr992)<br>G1	EGFR (Tyr1045)<br>H1	EGFR (Tyr1068)<br>I1
2	P1<br>A2	P2<br>B2	P3<br>C2	Blank<br>D2	Neg<br>E2	EGFR (Tyr845)<br>F2	EGFR (Tyr992)<br>G2	EGFR (Tyr1045)<br>H2	EGFR (Tyr1068)<br>I2
3	Blank<br>A3	Blank<br>B3	Blank<br>C3	Blank<br>D3	EGFR (Tyr1086)<br>E3	EGFR(Tyr1148)<br>F3	EGFR (Tyr1173)<br>G3	EGFR (Ser1046/1047)<br>H3	EGFR (Ser1070)<br>I3
4	Blank<br>A4	Blank<br>B4	Blank<br>C4	Blank<br>D4	EGFR (Tyr1086)<br>E4	EGFR(Tyr1148)<br>F4	EGFR (Tyr1173)<br>G4	EGFR (Ser1046/1047)<br>H4	EGFR (Ser1070)<br>I4
5	ErbB2 (Tyr877)<br>A5	ErbB2 (Tyr1112)<br>B5	ErbB2 (Tyr 1221/1222)<br>C5	ErbB2 (Tyr1248)<br>D5	ErbB2 (Thr686)<br>E5	ErbB2 (Ser1113)<br>F5	ErbB3 (Tyr1289)<br>G5	ErbB4 (Tyr1284)<br>H5	Blank<br>I5
6	ErbB2 (Tyr877)<br>A6	ErbB2 (Tyr1112)<br>B6	ErbB2 (Tyr 1221/1222)<br>C6	ErbB2 (Tyr1248)<br>D6	ErbB2 (Thr686)<br>E6	ErbB2 (Ser1113)<br>F6	ErbB3 (Tyr1289)<br>G6	ErbB4 (Tyr1284)<br>H6	Blank<br>I6
7	EGFR<br>A7	ErbB2<br>B7	ErbB3<br>C7	ErbB4<br>D7	Blank<br>E7	Blank<br>F7	Neg<br>G7	Blank<br>H7	P4<br>I7
8	EGFR<br>A8	ErbB2<br>B8	ErbB3<br>C8	ErbB4<br>D8	Blank<br>E8	Blank<br>F8	Neg<br>G8	Blank<br>H8	P4<br>I8

Supplementary Figure 13. **EGFR phosphorylation antibody arrays. A** EGFR-phosphorylation antibody arrays, with change in pan-EGFR chemiluminescence intensity (as measured in ImageJ) comparing $CuCl_2$ supplemented to unsupplemented lysates of the same biological replicate. In practice, subtle differences in chemiluminescence intensity for the EGFR phosphorylation sites were difficult to detect against the highly variable intensity of the membrane array background. **B** Key denoting antibody location identities from the arrays. P1-4 are reference (control) antibody spots. *n* = 3 biological replicates for each condition.

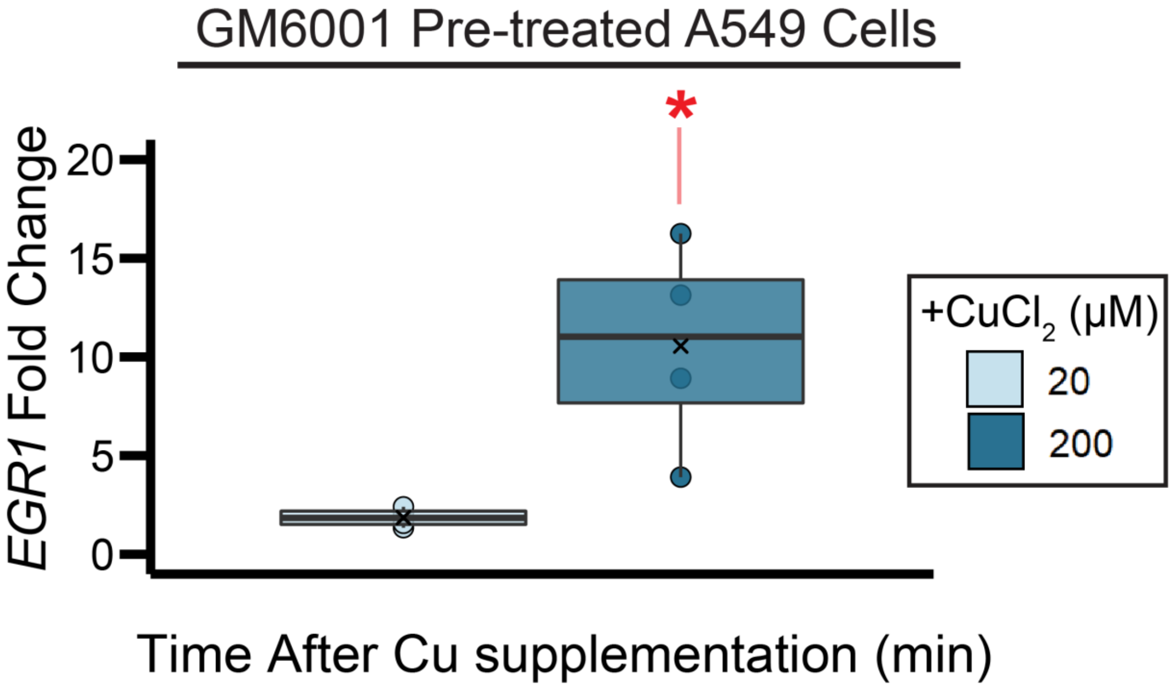

Supplementary Figure 14. **Effect of metalloproteinase inhibitor pretreatment on copper-stimulated changes in *EGR1* expression levels.** RNA-qPCR detected changes in *EGR1* transcript levels in RNA extracted from A549 cells following pretreatment with 20 µM GM6001 for 30 minutes and then $CuCl_2$ treatment for 60 minutes (relative to cells not supplemented with $CuCl_2$). Same box plot definitions as in Figure 1. *n* = 4 biological replicates for each condition. Source data are provided as a Source Data file, as are exact *p*-values.

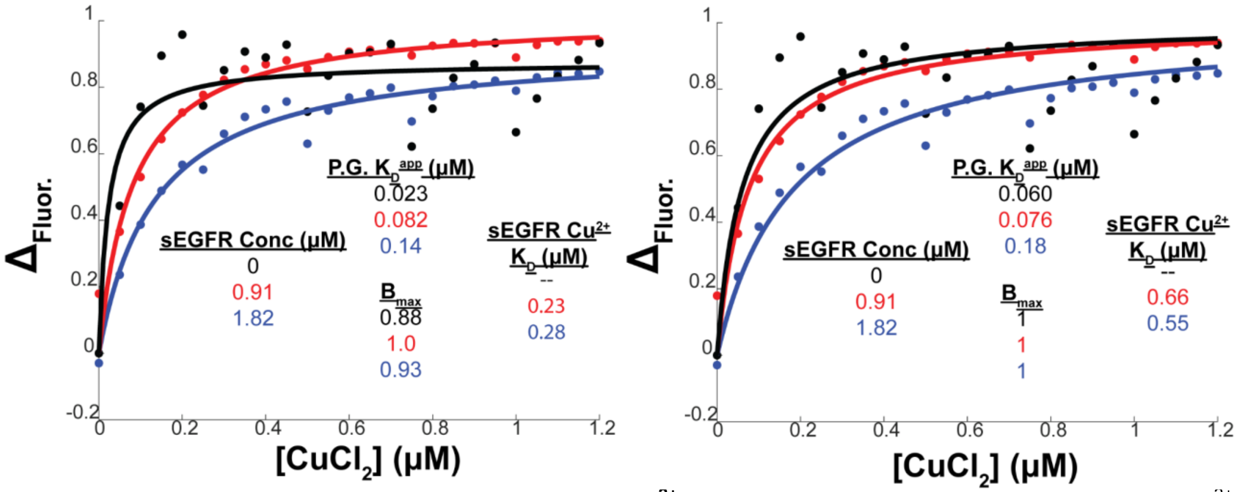

Supplementary Figure 15. **sEGFR and Phen Green Cu^{2+}-binding competition.** Fit to Phen Green Cu^{2+}-binding competition, (*left*) allowing B_{max} to float through the fitting procedure or **(***right*) fixing B_{max} at 1. See materials and methods for definitions of variables and methods for calculations. Importantly, we measured a Phen Green-Cu^{2+} K_D of 0.023-0.060 μM, very close to the previously reported value of 0.015 μM[67]. Definitions of affinity terms provided in the Materials and Methods.

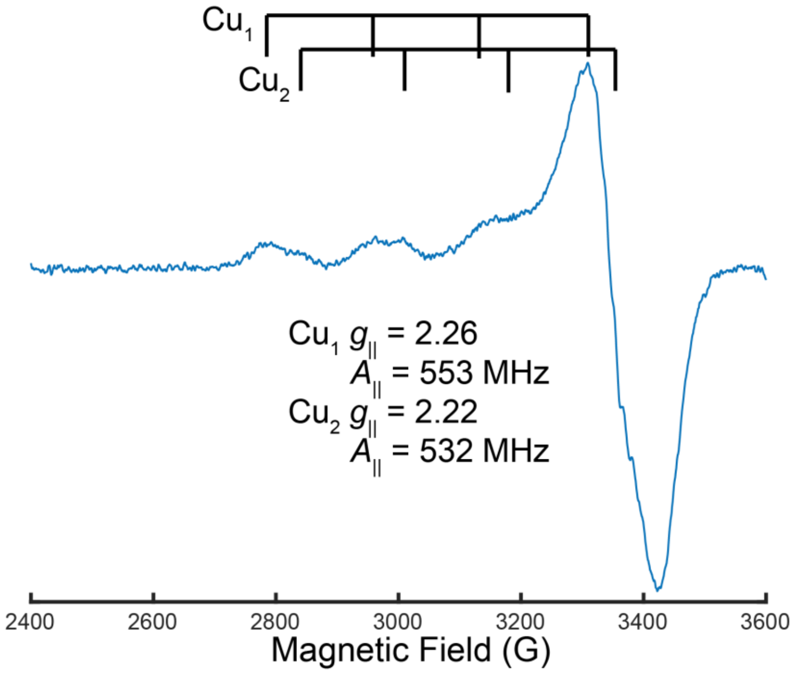

Supplementary Figure 16. **X-band EPR spectrum of sEGFR loaded with 1 Cu^{2+} equivalent.** The $g_{||}$ regions of the spectrum defines two well-structured Cu^{2+} coordination spheres, corresponding to either two distinct sites or one site with two slightly different ligation spheres. Collection conditions were as follows; 9.631 GHz microwave frequency, 30 dB microwave power attenuation, 9 G modulation amplitude, 100 kHz modulation frequency, 10.24 ms time constant, 84 s scan rate, 20 K temperature. Brackets denote the Cu^{2+} hyperfine splitting $A_{||}$.

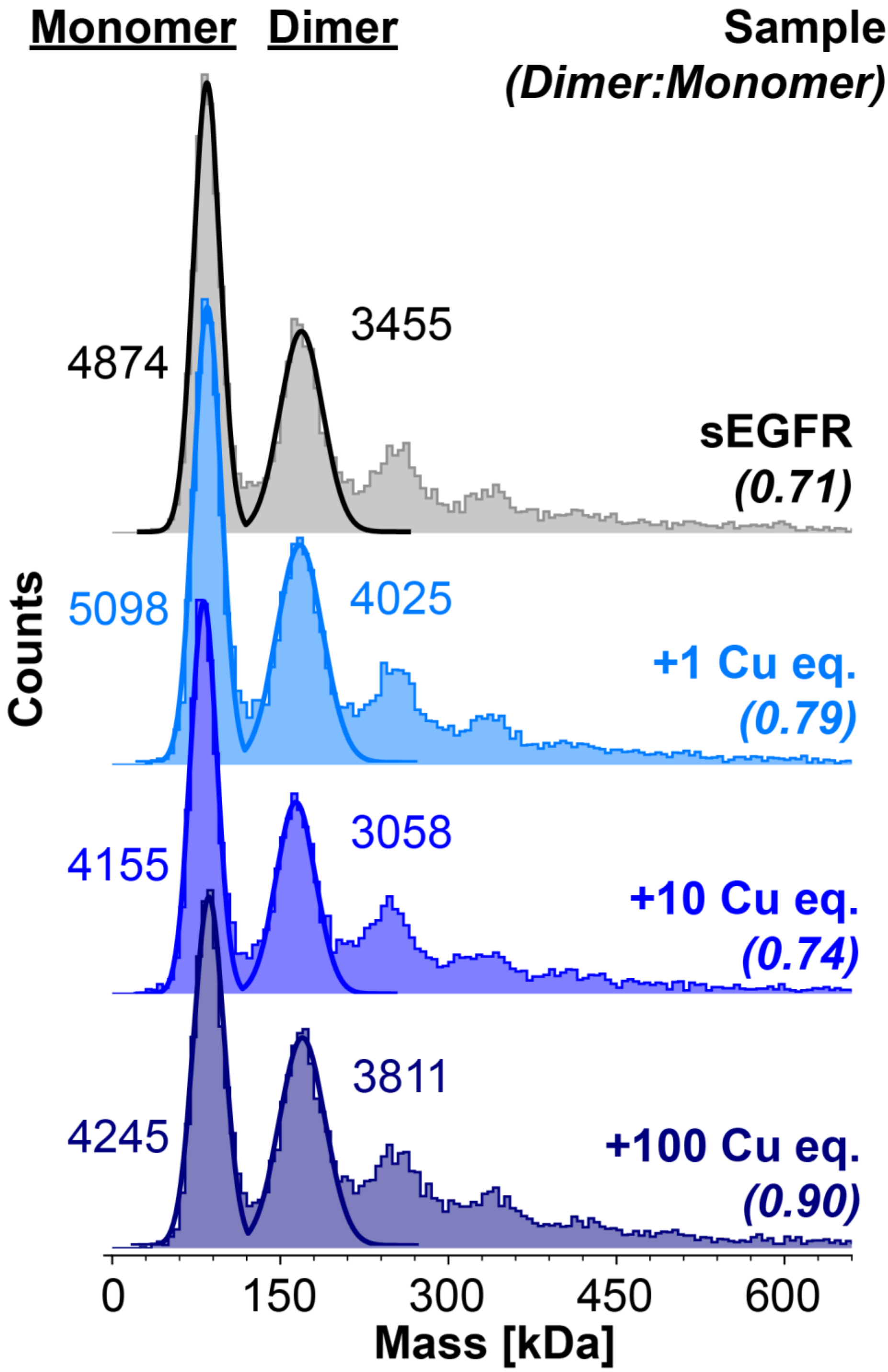

Supplementary Figure 17. **Mass photometry of sEGFR in the presence of increasing Cu concentrations.** Mass photometry of sEGFR (100 nM) incubated overnight (4℃) with various $CuCl_2$ equivalents. As the starting concentration of sEGFR was 100 nM, the 100 equivalent loaded sample corresponds to 10 μM Cu^{2+}, well above the experimentally-determined K_D (200-700 nM) of sEGFR for Cu^{2+}.

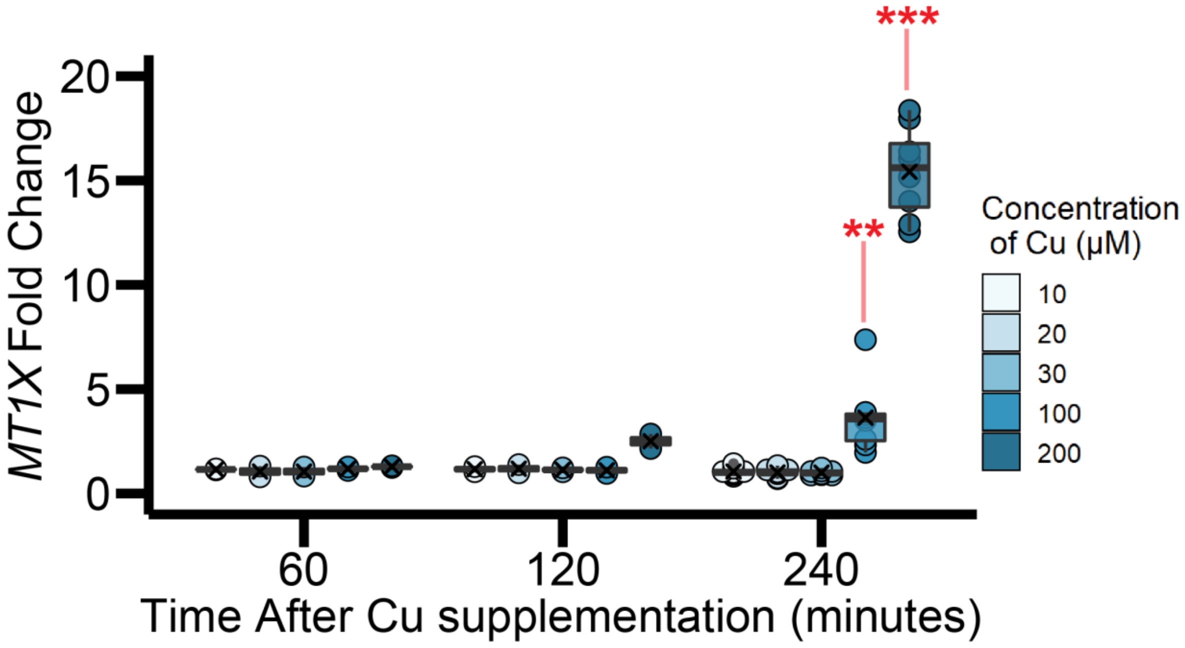

Supplementary Figure 18. **qPCR analysis of *MT1X* expression following Cu supplementation into A549 cells.** Asterisks denote standard meanings. n = 2, 2 and 8 biological replicates per Cu treatment for 60 min, 120 min, and 240 min post Cu supplementation, respectively. Same box plot definitions as in Figure 1. Source data are provided as a Source Data file, as are exact p-values.

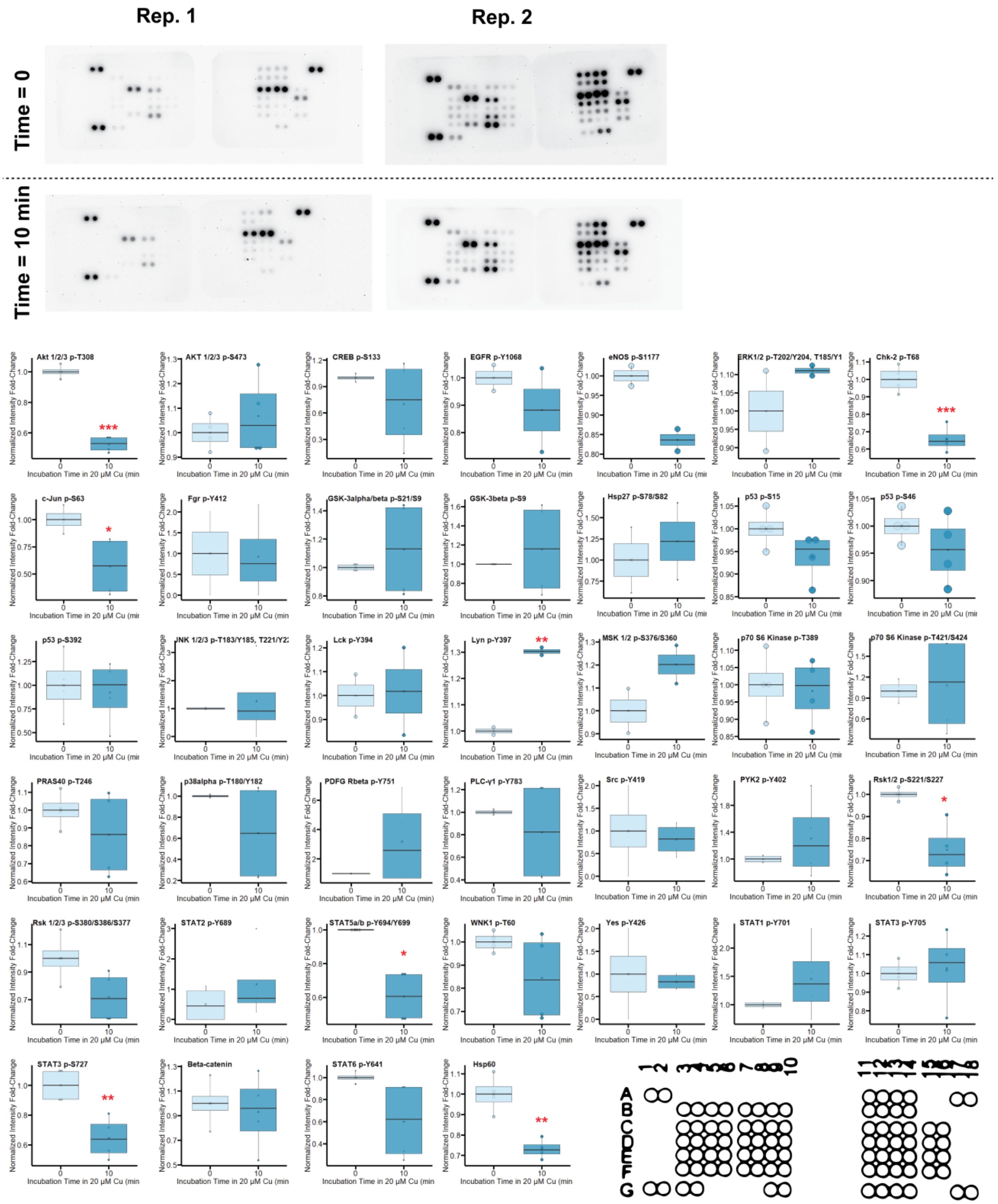

Supplementary Figure 19. **Proteome Profiler Antibody Arrays for HEK 293T cells treated with $ZnCl_2$ (20 µM).** The layout (and antibody spot locations) is the same as depicted in Supplementary Figure 3. *n* = 2 biological replicates, where each biological replicate contained antibodies spotted in duplicate. Same box plot definitions as in Figure 1.

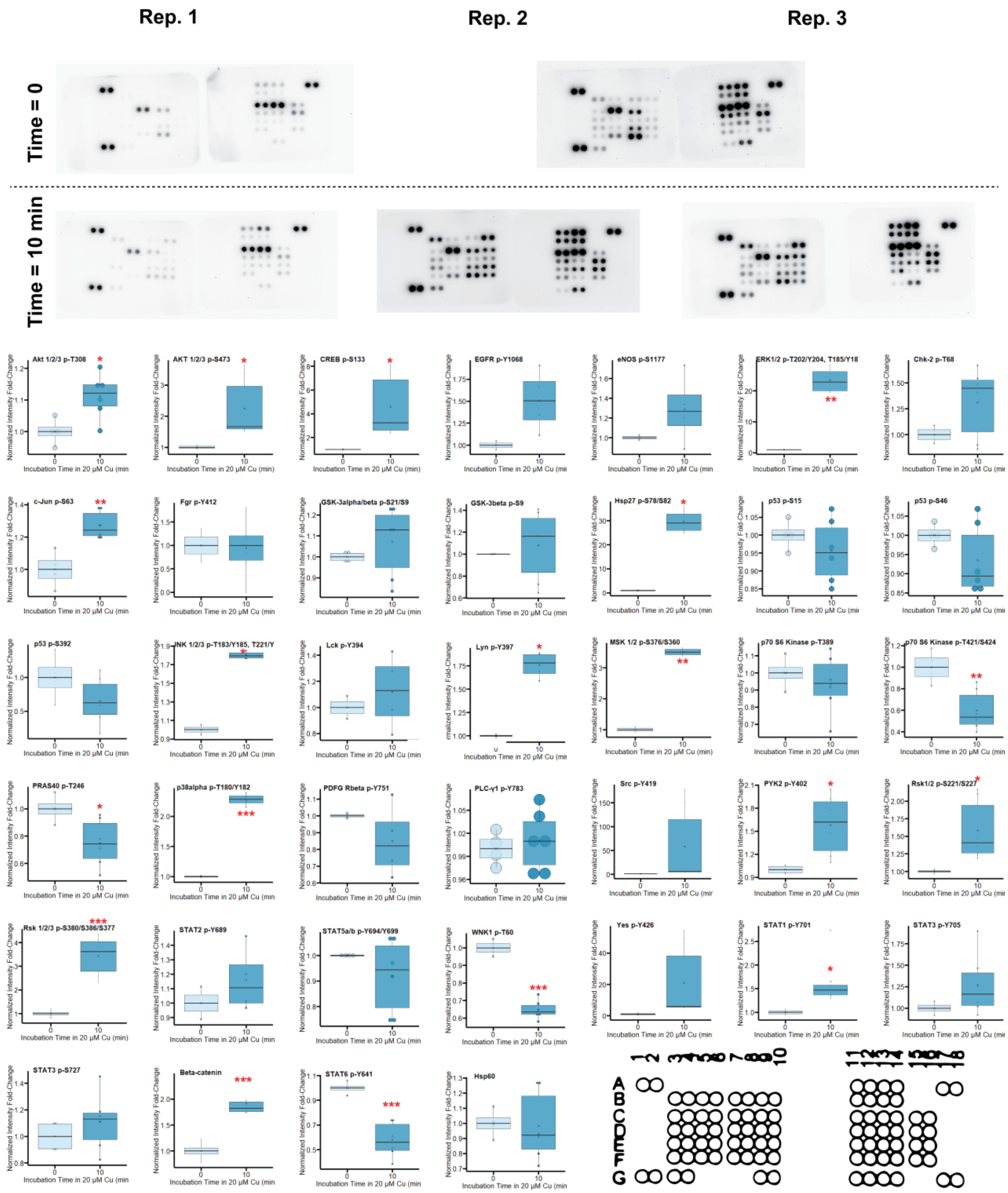

Supplementary Figure 20. **Proteome Profiler Antibody Arrays for HEK 293T cells treated with pervanadate (20 µM).** The layout (and antibody spot locations) is the same as depicted in Supplementary Figure 3. *n* = 2 and 3 biological replicates for t = 0 and t = 10 min, respectively, where each biological replicate contained antibodies spotted in duplicate. Same box plot definitions as in Figure 1. Time t = 0 replicate 2 is the same as Supplementary Fig. 19, as the experiments were performed in parallel, and therefore the control was the same for both.

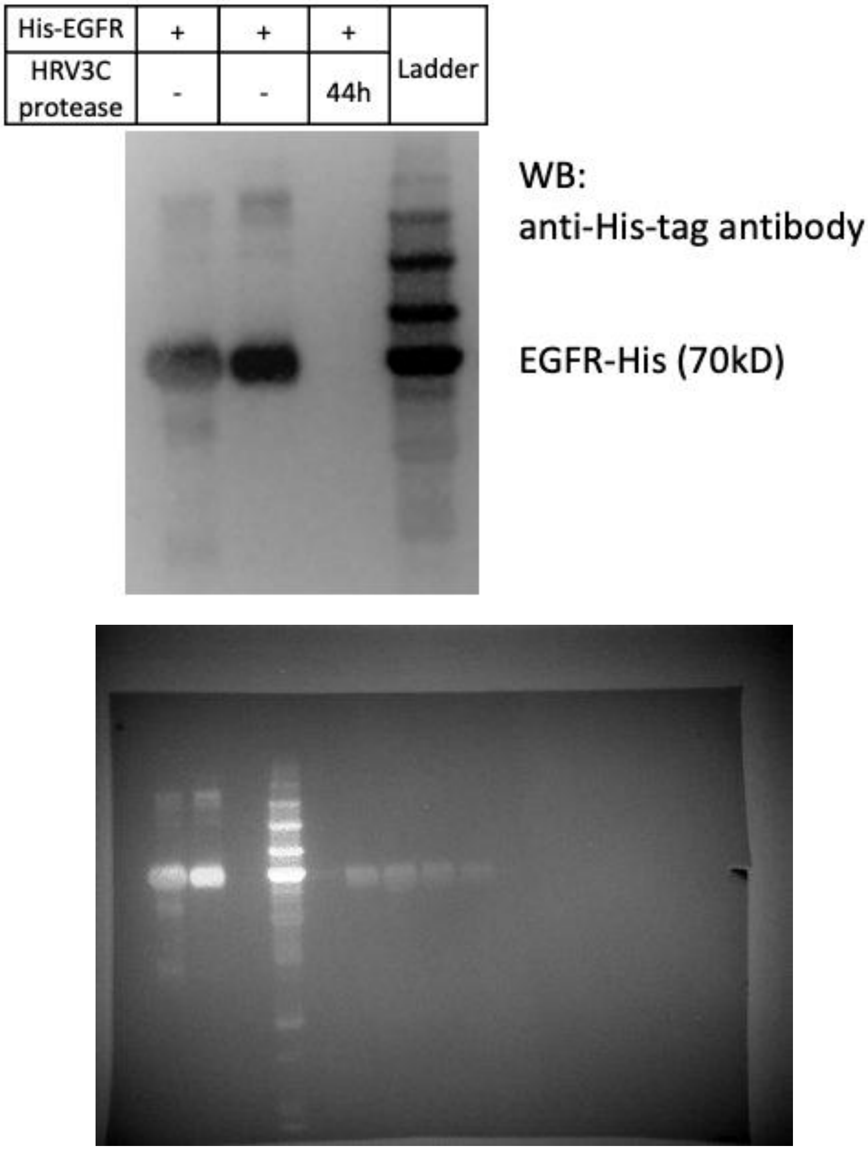

Supplementary Figure 21. **Western blot validation of His-tag removal from sEGFR construct (labeled His-EGFR).** Reaction conditions and protein solutions run on the gel are provided above the corresponding lanes. Uncropped, unedited image of full blot included on the bottom.

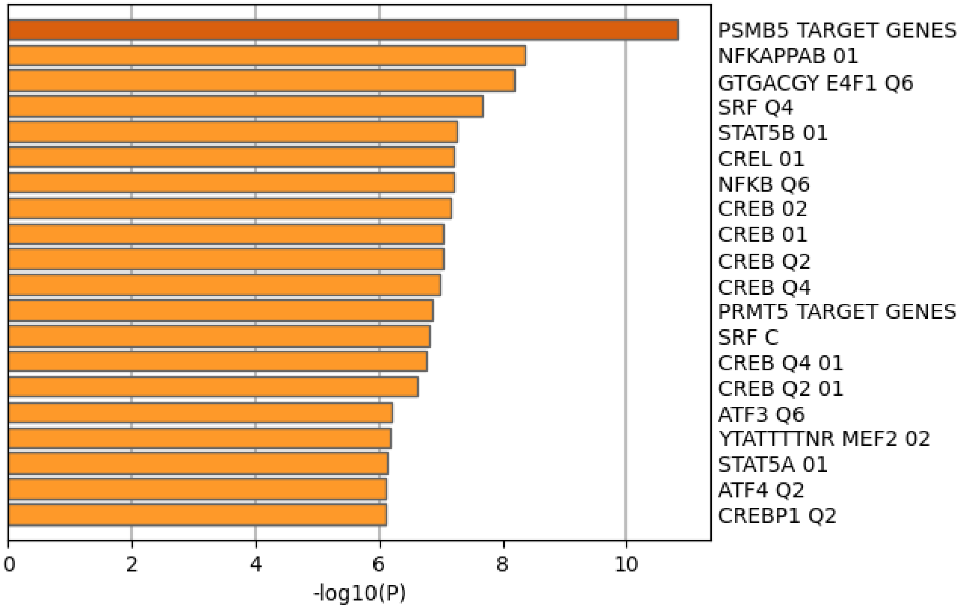

Supplementary Figure 22. **Fully-labelled TF enrichment generated from significantly differentially expressed transcripts post-30 μM $CuCl_2$ treatment.**

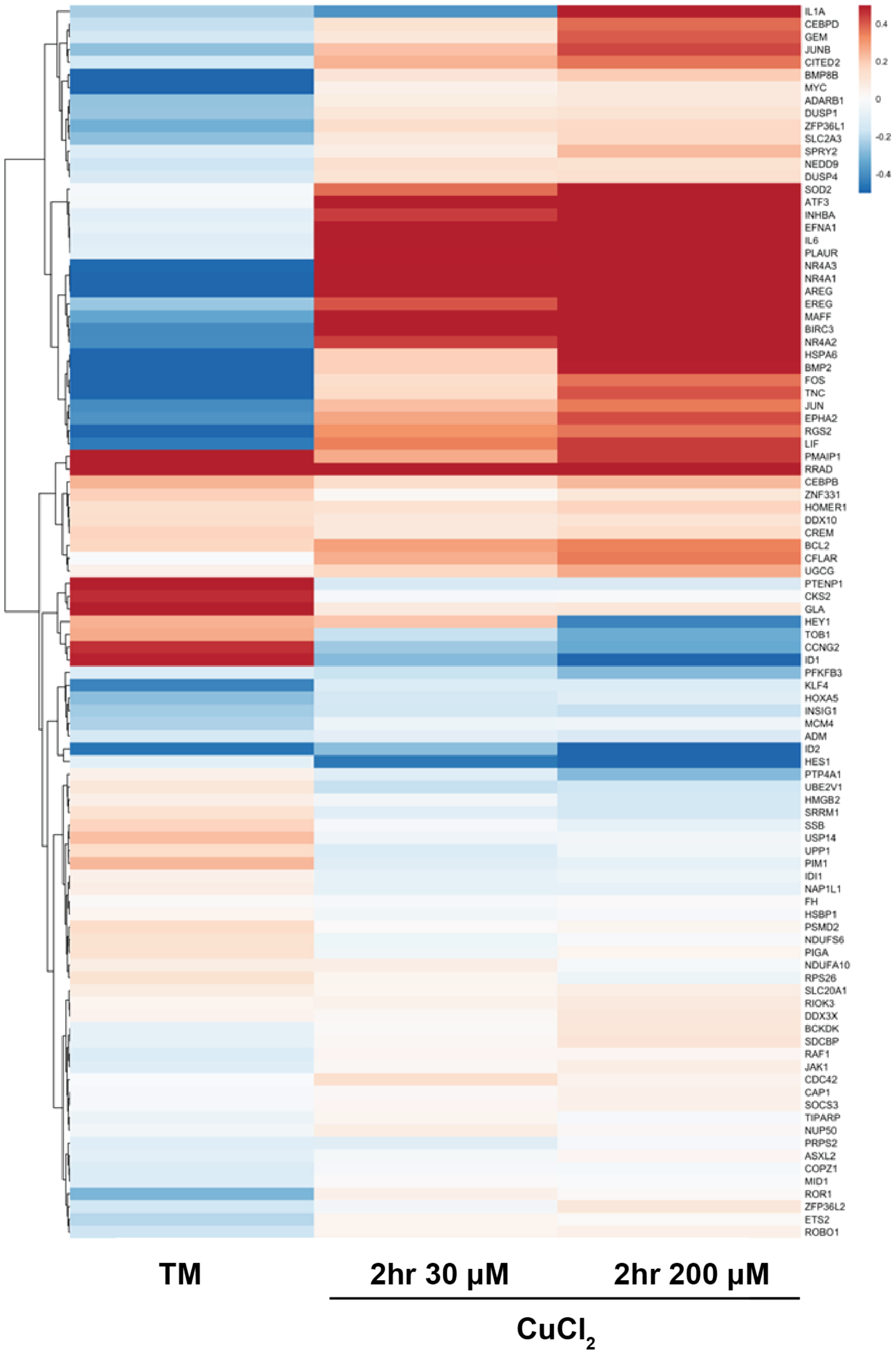
IL1A
CEBPD
GEM
JUNB
CITED2
BMP8B
MYC
ADARB1
DUSP1
ZFP36L1
SLC2A3
SPRY2
NEDD9
DUSP4
SOD2
ATF3
INHBA
EFNA1
IL6
PLAUR
NR4A3
NR4A1
AREG
EREG
MAFF
BIRC3
NR4A2
HSPA6
BMP2
FOS
TNC
JUN
EPHA2
RGS2
LIF
PMAIP1
RRAD
CEBPB
ZNF331
HOMER1
DDX10
CREM
BCL2
CFLAR
UGCG
PTENP1
CKS2
GLA
HEY1
TOB1
CCNG2
ID1
PFKFB3
KLF4
HOXA5
INSIG1
MCM4
ADM
ID2
HES1
PTP4A1
UBE2V1
HMGB2
SRRM1
SSB
USP14
UPP1
PIM1
IDI1
NAP1L1
FH
HSBP1
PSMD2
NDUFS6
PIGA
NDUFA10
RPS26
SLC20A1
RIOK3
DDX3X
BCKDK
SDCBP
RAF1
JAK1
CDC42
CAP1
SOCS3
TIPARP
NUP50
PRPS2
ASXL2
COPZ1
MID1
ROR1
ZFP36L2
ETS2
ROBO1
0.4
0.2
0
-0.2
-0.4
TM
2hr 30 µM
2hr 200 µM
CuCl2

Supplementary Figure 23. **CREB1-target gene hierarchical cluster.** Hierarchical cluster of differential expression of CREB1-target genes in the TM-treated and 2 hr $CuCl_2$-treated RNA-seq results. List of CREB1-target genes was generated from the human cell line cAMP-responsive genes in [34] – of the 195 human cAMP-responsive genes, 97 produced responses following DESeq2 analysis in TM and copper conditions, as shown in the hierarchical cluster above.

Target Gene	HEK CREB (Binding Ratio, *p*-value)	Hepatocyte CREB (Binding Ratio, *p*-value)	Hepatocyte p-CREB (Binding Ratio, *p*-value)	Islet p-CREB (Binding Ratio, *p*-value)	HEK p-CREB 0h 1h 4h (Binding Ratio, *p*-value)	CRE prediction
CTR1	3.8, 2.50E-09	2.1, 4.80E-05	3.6, 5.60E-07	1.1, 1.50E-01	2.6, 3.50E-07<br>3.9, 3.60E-11<br>3.5, 6.40E-09	CRE_NoTATA

Supplementary Table 1. **CREB Target Gene Database results for human *SLC31A1* (*CTR1*)**. CREB or p-CREB (phospho-CREB) indicates the ChIP target for ChIP-seq experimental results reported below. “0h”, “1h”, and “4h” for HEK samples indicate duration of forskolin stimulation (to generate cAMP). CRE prediction value "CRE_NoTATA" indicates a predicted functional CRE without the presence of a proximal TATA box. Data accessed 6/18/22.

Target Gene	TF	Knock-Method	Tissue Type	Biosample	Fold-Change	*p*-value	FDR-q	Dataset ID
CTR1	CREB1	shRNA	Haematopoietic and lymphoid tissue	K562	1.55975	1.46E-07	2.45E-05	DataSet_01_004
CTR1	CREB1	shRNA	Parotid gland	H3118 MEC	0.60725	1.01E-02	1.84E-01	DataSet_01_194
CTR1	CREB1	shRNA	Parotid gland	HSY	0.61149	5.12E-03	3.20E-01	DataSet_01_195

Supplementary Table 2**. KnockTF results assessing *SLC31A1*/*CTR1* expression level changes induced by CREB1 knockdown.** Statistically-significant (FDR-q < 0.05) change measured in K562 cells shown in yellow, with statistically-insignificant results shown in gray.

Tissue	Transcript	Pearson's Correlation Coeff. (R)	*p*-value
Lung	*EGR1*	-0.093	0.025
	NAB1	-0.51	< 2.2e-16
	NAB2	-0.25	8.1e-10
	NR4A1	-0.24	4.6e-09
	CREB1	-0.20	9.5e-07
Heart	*EGR1*	-0.22	1.1e-10
	NAB1	-0.49	< 2.2e-16
	NAB2	-0.19	2.1e-8
	NR4A1	-0.29	< 2.2e-16
	CREB1	-0.017	0.624
Brain	*EGR1*	-0.027	0.17
	NAB1	-0.35	< 2.2e-16
	NAB2	-0.41	< 2.2e-16
	NR4A1	0.0062	0.75
	CREB1	-0.15	1.8e-15
Liver	*EGR1*	-0.089	0.18
	NAB1	0.017	0.80
	NAB2	-0.19	0.0051
	NR4A1	-0.15	0.028
	CREB1	-0.14	0.034
Kidney	*EGR1*	-0.18	0.099
	NAB1	-0.52	1.9e-7
	NAB2	0.14	0.19
	NR4A1	-0.085	0.43
	CREB1	-0.45	9.09e-06

Supplementary Table 3. **GTEx transcript expression level correlations with *CTR1* expression levels.** Statistically-insignificant correlations shown in gray. Two-sided Pearson correlation *p*-values reported in right column.

**Transcript**	**Forward Primer (5' → 3')**	**Reverse Primer (5' → 3')**
ATOX1	CTGTGGAGGCTGTGCTGAAG	TCTTGTTGGGCAGGTCAATG
CTR1	TCGCTACAATTCCATGCCTGTC	TTGCAGGAGGTGAGGAAAGC
CREB1	CGAGAACCAGCAGAGTGGAG	GGACTTGAACTGTCTGCCCA
EGR1	AGCCCTACGAGCACCTGAC	GGGCAGTCGAGTGGTTTG
GAPDH	CCCACTCCTCCACCTTTGAC	CCACCACCCTGTTGCTGTAG
NAB1	TGCAGGGCTTTACAGGCAGAG	AGGCATTCGGAGATTCAAAGGTC
NAB2	AGATCTCTGAGACTGCGGGT	GGGCTCTTGGGGCTAAAACT
MT1X	ACCACGCTTTTCATCTGTCC	GAGCAGTTGGGGTCCATTTC
DMT1	AGCAGGAAGTTCGAGAAGCC	AAAGAGGCCAGCATGAGGAC

Supplementary Table 4. **Primers used for RNA-qPCR experiments.**
