# Supplementary material for: PTPN2 copper-sensing relays copper level fluctuations into EGFR/CREB activation and associated CTR1 transcriptional repression
Source: Nat Commun. 2024 Aug 13;15:6947. doi: 10.1038/s41467-024-50524-5 (PMC11322707; doi:10.1038/s41467-024-50524-5)
Supplement: Supplementary file 3 — Description of Additional Supplementary Files [file 41467_2024_50524_MOESM3_ESM.pdf]

Description of Additional Supplementary Files

File Name: Supplementary Data 1
Description: DESeq2-generated differential expression data generated from A549 cells treated with Cu vs. untreated cells (KAS-seq experiment). pvalue denotes twosided Wald test as calculated by DESeq2, without correction for multiple observations, while padj is the false-discovery rate adjusted p-value.

File Name: Supplementary Data 2
Description: DESeq2-generated differential expression data generated from MDA-MB-468 cells treated with TM vs. DMSO-treated cells. pvalue denotes two-sided Wald test as calculated by DESeq2, without correction for multiple observations, while padj is the false-discovery rate adjusted pvalue.

File Name: Supplementary Data 3
Description: DESeq2-generated differential expression data generated from A549 cells treated with Cu vs. untreated cells (RNA-seq experiment). pvalue denotes twosided Wald test as calculated by DESeq2, without correction for multiple observations, while padj is the false-discovery rate adjusted p-value.

File Name: Supplementary Data 4
Description: Gene ontology enrichment terms, with statistical parameters, generated from differential expression data from MDA-MB468 RNA-seq experiments, comparing cells treated with TM to DMSO-treated control cells. pvalue were calculated via two- sided Fisher's exact test, uncorrected for multiple observations. q-value corresponds to the false discovery rate corrected p-value.

File Name: Supplementary Data 5
Description: Gene ontology enrichment terms, with statistical parameters, generated from differential expression data from A549 RNAseq experiments, comparing cells treated with 30 uM $CuCl_2$ for 2 hrs to untreated control cells. pvalue were calculated via twosided Fisher's exact test, uncorrected for multiple observations. q-value corresponds to the false discovery rate corrected p-value.
