# Supplementary material for: PTPN2 copper-sensing relays copper level fluctuations into EGFR/CREB activation and associated CTR1 transcriptional repression
Source: Nat Commun. 2024 Aug 13;15:6947. doi: 10.1038/s41467-024-50524-5 (PMC11322707; doi:10.1038/s41467-024-50524-5)
Supplement: Supplementary file 9 — Reporting Summary [file 41467_2024_50524_MOESM9_ESM.pdf]

nature portfolio

Corresponding author(s): Matthew Ross, Chuan He

Last updated by author(s): May 26, 2024

Reporting Summary

Nature Portfolio wishes to improve the reproducibility of the work that we publish. This form provides structure for consistency and transparency in reporting. For further information on Nature Portfolio policies, see our Editorial Policies and the Editorial Policy Checklist.

Statistics

For all statistical analyses, confirm that the following items are present in the figure legend, table legend, main text, or Methods section.

n/a	Confirmed	
☐	☒	The exact sample size (*n*) for each experimental group/condition, given as a discrete number and unit of measurement
☐	☒	A statement on whether measurements were taken from distinct samples or whether the same sample was measured repeatedly
☐	☒	The statistical test(s) used AND whether they are one- or two-sided<br>*Only common tests should be described solely by name; describe more complex techniques in the Methods section.*
☒	☐	A description of all covariates tested
☐	☒	A description of any assumptions or corrections, such as tests of normality and adjustment for multiple comparisons
☐	☒	A full description of the statistical parameters including central tendency (e.g. means) or other basic estimates (e.g. regression coefficient) AND variation (e.g. standard deviation) or associated estimates of uncertainty (e.g. confidence intervals)
☐	☒	For null hypothesis testing, the test statistic (e.g. *F*, *t*, *r*) with confidence intervals, effect sizes, degrees of freedom and *P* value noted<br>*Give P values as exact values whenever suitable.*
☒	☐	For Bayesian analysis, information on the choice of priors and Markov chain Monte Carlo settings
☒	☐	For hierarchical and complex designs, identification of the appropriate level for tests and full reporting of outcomes
☐	☒	Estimates of effect sizes (e.g. Cohen's *d*, Pearson's *r*), indicating how they were calculated

Our web collection on statistics for biologists contains articles on many of the points above.

Software and code

Policy information about availability of computer code

Data collection	No commercial, open source, or custom code was used for data collection
Data analysis	Antibody arrays: ImageJ 1.53k<br>ChIP-seq: Wiggletools v1.2.11, ucsc-wigtobigwig (v377)<br>RNA-qPCR: dplyr v1.0.7, tidyverse v1.3.1, readxl v1.3.1, ggprism v1.0.3<br>KAS-seq: Used KAS-pipe (dependencies with versions provided at https://github.com/Ruitulyu/KAS-pipe)<br>MDA-MB-468 RNA-seq: KAS-pipe for adapter trimming, STAR version 2.7.9a, DESeq2 v1.36.0, GSEA v4.2.3<br>A549 RNA-seq: fastqc v0.11.9, trim-galore v0.6.7, HISAT2 v2.2.1, Samtools v1.13, DESeq2 v1.34.0<br>GTEx: DESeq2 v1.36.0, ggpubr v0.4.0

For manuscripts utilizing custom algorithms or software that are central to the research but not yet described in published literature, software must be made available to editors and reviewers. We strongly encourage code deposition in a community repository (e.g. GitHub). See the Nature Portfolio guidelines for submitting code & software for further information.

Data

Policy information about availability of data

All manuscripts must include a data availability statement. This statement should provide the following information, where applicable:

- Accession codes, unique identifiers, or web links for publicly available datasets
- A description of any restrictions on data availability
- For clinical datasets or third party data, please ensure that the statement adheres to our policy

The high throughput sequencing data generated in this study have been deposited in the Gene Expression Omnibus (GEO), accession GSE211339 [https://www.ncbi.nlm.nih.gov/geo/query/acc.cgi?acc=GSE211339] (A549 KAS-seq), GSE210777 [https://www.ncbi.nlm.nih.gov/geo/query/acc.cgi?acc=GSE210777] (MDA-MB-468 RNA-seq), and GSE214566 [https://www.ncbi.nlm.nih.gov/geo/query/acc.cgi?acc=GSE214566] (A549 RNA-seq). Metascape gene ontology (GO) enrichment analysis summaries are provided as Supplementary Tables 4 (MDA-MB-468 TM-treated) and 5 (2 hour, 30 µM CuCl2 treated A549 RNA-seq). DESeq2 differential expression analysis results from the various KAS-seq and RNA-seq experiments are provided as Supplementary Tables 7 (A549 KAS-seq), 8 (MDA-MB-468 RNA-seq), and 9 (A549 RNA-seq). Source data are provided with this paper. Specifically, raw qPCR data (fold-changes), ICP-MS Cu-stoichiometries, and pNPP Assay absorbance readings are included in the Source Data file. Raw mass photometry movies are available under restricted access due to file size limitations, access can be obtained by contacting the corresponding authors. GTEx and TCGA RNA-seq data were accessed from, and are available via, https://gtexportal.org/home/ and https://xenabrowser.net/, respectively. ChIP-Atlas ChIP-seq data are available via https://chip-atlas.org/. CREB target gene database data is available via http://signal.salk.edu/creb/index.html.

Research involving human participants, their data, or biological material

Policy information about studies with human participants or human data. See also policy information about sex, gender (identity/presentation), and sexual orientation and race, ethnicity and racism.

Reporting on sex and gender	No human participants were involved. Sex and gender were not considered in the assessment of human immortalized cell lines, as no sex-/gender-identical cell lines exist for immortalized cell lines under consideration. Thus, assessing both male and female derived immortalized cell lines would necessarily also come with changes to critical signaling pathway activation of the cell lines.
Reporting on race, ethnicity, or other socially relevant groupings	No human participants were involved
Population characteristics	No human participants were involved
Recruitment	No human participants were involved
Ethics oversight	No human participants were involved

Note that full information on the approval of the study protocol must also be provided in the manuscript.

Field-specific reporting

Please select the one below that is the best fit for your research. If you are not sure, read the appropriate sections before making your selection.

☒ Life sciences ☐ Behavioural & social sciences ☐ Ecological, evolutionary & environmental sciences

For a reference copy of the document with all sections, see nature.com/documents/nr-reporting-summary-flat.pdf

Life sciences study design

All studies must disclose on these points even when the disclosure is negative.

Sample size	For all experiments except RNA-qPCR, experimental sample sizes were pre-determined to be performed in triplicate (or duplicate in the case of KAS-seq) so as to be able to evaluate statistical significance and identify any outlier/anomalous replicates. KAS-seq experiments were performed in duplicate due to the cost-prohibitive nature of the overall experiment. These sample sizes are both sufficient to run advanced analysis (e.g. GSEA on RNA-seq samples), while minimizing false positives in the KAS-seq data (e.g. only two "hits" at 15 min post Cu-supplementation). The RNA-qPCR sample sizes were initially envisioned as two biological replicates at each condition (enough for detection of dose-response and statistical significance in changes), but as the experiment evolved and transcripts of interest were added/removed, the previously chosen transcripts (EGR1, CTR1, NAB1, and NAB2) were repeatedly evaluated as well. In the case of RNA-qPCR results, the replicates are enough for both statistical significance and to identify trends (such as dose-response) across the Cu supplementation concentrations. Additionally, more biological replicates were performed in response to reviewer comments during a prior journal submission. No statistical method was predetermined before-hand to define sample sizes.
Data exclusions	1 out of 2 total biological replicates each of NAB1 and NAB2 RNA-qPCR (0-200 µM CuCl2, 60 min incubation) were excluded for being anomalous; the replicate fold-changes were >3 standard deviations above the mean. 1 out of 22 total biological replicates of CTR1 (30-100 uM CuCl2, 4 hr incubation) was excluded for being anomalous; the biological replicate fold-changes were >3 standard deviations above the mean for multiple transcript conditions. The exclusion criteria were not pre-established. Inclusion of the NAB1, NAB2, and CTR1 100 µM CuCl2

data points would not affect any conclusions in the manuscript. Inclusion of the CTR1 4 hr incubation 30 μM $CuCl_2$ would result in the 4 hr, 30 μM $CuCl_2$ CTR1 fold-change increase being statistically significant but would otherwise not affect any of the conclusions in the manuscript.

Replication: All replicates used to derive our conclusions are reported in the manuscript, and were successful in validating the conclusions stated therein.

Randomization: This is not relevant to the study; treated and untreated cells were derived from the same cell suspension immediately after splitting.

Blinding: Blinding was not possible, as the person treating the cells (or leaving them untreated) was generally the same individual isolating DNA/RNA and performing downstream experiments with the isolated DNA/RNA.

Reporting for specific materials, systems and methods

We require information from authors about some types of materials, experimental systems and methods used in many studies. Here, indicate whether each material, system or method listed is relevant to your study. If you are not sure if a list item applies to your research, read the appropriate section before selecting a response.

Materials & experimental systems

n/a	Involved in the study	
☐	☒	Antibodies
☐	☒	Eukaryotic cell lines
☒	☐	Palaeontology and archaeology
☒	☐	Animals and other organisms
☒	☐	Clinical data
☒	☐	Dual use research of concern
☒	☐	Plants

Methods

n/a	Involved in the study	
☒	☐	ChIP-seq
☒	☐	Flow cytometry
☒	☐	MRI-based neuroimaging

Antibodies

Antibodies used: Mouse anti-His-tag monoclonal antibody (Genscript, A00186-100, clone ID 6G2A9) was used at a final concentration of 0.5 µg/mL

Validation: No validation mentioned on the Genscript website for this antibody

Eukaryotic cell lines

Policy information about cell lines and Sex and Gender in Research

Cell line source(s): ATCC

Authentication: A549 cells (catalog number: A549 (ATCC® CCL-185™))were purchased directly from ATCC, HEK 293T were acquired from a lab member who acquired them from ATCC. Neither was independently authenticated.

Mycoplasma contamination: All cell lines tested mycoplasma negative with MycoProbe® Mycoplasma Detection Kit

Commonly misidentified lines (See ICLAC register): No commonly misidentified cell lines were used in the study
